# Hypertension and Valvular Heart Disease: An Overview of a Complex and Clinically Meaningful Relationship

**DOI:** 10.3390/life16010125

**Published:** 2026-01-14

**Authors:** Roxana Oana Darabont, Diana Mihalcea, Dragos Vinereanu

**Affiliations:** 1 Cardiology and Cardiovascular Surgery Department, “Carol Davila” University of Medicine and Pharmacy, 020021 Bucharest, Romania; rdarabont@yahoo.com (R.O.D.); vinereanu@gmail.com (D.V.); 2 University and Emergency Hospital of Bucharest, 050098 Bucharest, Romania

**Keywords:** hypertension, valvular heart disease, aortic stenosis, aortic regurgitation, mitral stenosis, mitral regurgitation

## Abstract

The overlooked relationship between hypertension (HTN) and valvular heart disease (VHD) has been brought to wider attention by large-scale population studies and the latest guidelines. Approximately 45% of patients with VHD have been diagnosed with HTN. This association increases with age, but cannot be explained solely by the rising prevalence of both conditions. HTN promotes the onset or worsening of VHD by mechanically stressing the valves and by modified blood flow dynamics through increased filling pressures and afterload. It has been shown that a 20 mmHg rise in BP triples the risk of aortic valvular disease and mitral regurgitation. This review will address the impact of HTN on the occurrence and progression of valvular lesions, its effect on the prognosis of patients with VHD, and the available data on blood pressure management. As long as relatively well-documented data on aortic valve disease are available, studies are still needed to clarify target blood pressure values under therapy and the most appropriate drug classes for mitral regurgitation, especially in its primary forms.

## 1. Introduction

The overlooked relationship between hypertension (HTN) and valvular heart disease (VHD) is being brought to wider attention by large-scale population studies [1,2,3,4,5], as well as in the latest HTN management guidelines in Europe and the USA [6,7]. Nearly 60% of patients with VHD have at least one of four common cardiovascular (CV) risk factors, including high blood pressure (BP) [5.6]. Approximately 45% of patients with VHD have been diagnosed with HTN [5,6]. In addition, Valvular Heart Disease II Survey identifies high BP as the leading modifiable CV risk factor in VHD patients, with a prevalence of 76% in aortic stenosis (AS), 61% in aortic regurgitation (AR), 42% in mitral stenosis (MS), and 56% and 68% in primary and secondary mitral regurgitation (MR), respectively [8]. This relationship increases with age but cannot be explained solely by the rising prevalence of both conditions over recent decades, driven by population aging [9]. It is, in fact, a complex interaction, as BP strongly influences the function of the heart valves. A previous study showed that a 20 mmHg increase in systolic BP or a 15 mmHg increase in pulse pressure (PP) triples the risk of aortic valve disease and mitral valve regurgitation [5]. Therefore, HTN can accelerate the progression of VHD by exerting direct mechanical stress on the valves, resulting in damage to the valve leaflets and dilatation of the valve annulus. In addition, high BP contributes to remodeling of the left atrium (LA) and left ventricle (LV), increasing LV filling pressure and influencing both preload and afterload, with consequences for blood flow through valvular lesions [2,3,4].

The reported prevalence of VHD is also influenced by a country’s level of economic development and the criteria used to define valvular lesions. In economically underdeveloped countries, rheumatic valvular abnormalities predominate [10]. On the other hand, in economically developed countries, VHD is mainly caused by degenerative processes related to aging and CV risk factors, which, according to some authors, will trigger “a tsunami” of degenerative VHD [10,11]. Major epidemiological studies, which were mainly conducted in the elderly, have found a prevalence of VHD ranging from 28% to 51% [12,13]. In most cases, the reported valvular lesions were mild [12,13]. Notably, in older patients, even mild lesions can significantly impact outcome. An echocardiographic study showed that the incidence of mitral valve dysfunction increased by 10% over 10 years in patients with baseline mitral valve annulus calcification [14]. In the Atherosclerosis Risk in Communities study, the prevalence of severe valve lesions increased from 1% to 7% over 6 years, worsening the prognosis in these individuals [15].

There are no large randomized controlled trials examining the role of antihypertensive medications in the progression of various VHDs; however, some observational data may help optimize hypertension treatment in these patients. In this review, we summarize the role of hypertension in the incidence and progression of valvular lesions, its prognostic impact, and the available therapeutic options.

Therefore, we conducted a systematic literature search in PubMed. The key words used were “hypertension”, “arterial hypertension”, valvular heart disease”, “aortic stenosis”, “aortic regurgitation”, “mitral stenosis”, and “mitral regurgitation”. Studies about epidemiology, clinical features, imaging and biological data, prognosis, and therapeutic options in patients with concomitant VHD and HTN were included.

## 2. Aortic Stenosis

Aortic valve stenosis is the most common valvular disease in economically developed countries, with prevalence increasing with age and the presence of atherosclerotic disease. According to epidemiological data, AS occurs in 2.6% of individuals aged 75 years or older [16,17,18]. Of note, 76–78% of patients with degenerative aortic lesions also have high BP [17]. HTN increases by 23% the risk of aortic valve calcification, along with other known factors such as smoking, obesity, hyperlipidemia, and diabetes [18,19]. Arterial stiffness is often increased in this patient group, amplifying afterload through elevated central systolic BP and outflow impedance [17]. A summary of clinical trials with HTN prevalence in AS patients is presented in Table 1.

Usually, the LV ejection fraction (EF) is maintained for a prolonged period in patients with AS, even in patients with severe lesions [17]. LV remodeling, caused by high systolic wall stress and thereby disturbed myocardial perfusion, promotes the development of subendocardial stress and the formation of secondary reactive fibrosis [17]. When AS coexists with HTN, a maladaptive LV response results in additional wall thickening, mechanical myocardial desynchronization, and worsening diastolic function [20]. Using tissue Doppler imaging and 2-dimensional speckle-tracking transthoracic echocardiography, subclinical LV dysfunction can be detected even when EF remains normal. A myocardial velocity of the septal mitral annulus less than 5.4 cm/s or a global longitudinal strain (GLS) lower than −15% has been shown to correlate with a poor prognosis in these patients [21,22].

**Table 1:** Summary of clinical studies that analyzed the prevalence of hypertensive in patients with aortic stenosis.

Study	Type of Study	Number of Subjects	Number of AS Patients	AS Prevalence (%)	HTN Prevalence in AS Patients (%)
Rieck et al. [1]	Echocardiographic	1616	1616	100	82
Tasted et al. [3]	Multidetector CT	101	101	100	37
Rahimi et al. [4]	Cohort—general population	5,392,000	20,680	0.38	Each 20 mmHg increment in systolic BP associates with risk of AS [OD 1.41; 95% CI 1.38–1.45]
Nazarzadeh et al. [5]	Cohort—general population	502,602	1491	0.45	Each 20 mmHg increment in systolic BP associates with risk of AS [OD 3.26; 95% CI 1.5–7.1]
Iung et al. [8]	Prospective—severe VHD	7247	2152	29	76.5
Lu et al. [9]	Prospective—moderate and severe VHD	11,862	568	0.04	20.4 39 (AS + AR)
Tsampasian et al. [12]	Cohort—general population	4237	44	1	40
D’Arcy et al. [13]	Cohort- general population > 65 years	2500	32	1.3 Mild calcific valve 34 Significant calcific valve 0.7	54.8 in all VHD
Shelbaya et al. [15]	Prospective community-based cohort study	6118	missing	15.4 stage A 4.1 stage B 0.4 stage C 0.4 stage D 32 pts surgical valve history	85 stage A of all VHD 86 stage B of all VHD 88 stage C/D of all VHD
Wang et al. [19]	General population 50–64 years—cardiac CT	29,221	2053—aortic valve calcification	0.07	37
Nielsen et al. [23]	Patients with asymptomatic AS and no symptomatic atherosclerotic disease	1767	1767	100	55

AR: aortic regurgitation; AS: aortic stenosis; BP: blood pressure; CT: computer tomography; HTN: hypertension; VHD: valvular heart disease.

Both severe AS and HTN can induce LV hypertrophy and manifestations of heart failure with preserved EF. In medical practice, it would be helpful to distinguish the contributions of these pathological conditions to LV remodeling and dysfunction or to evaluate the amplifying effect of HTN on the AS. This would allow for the development of personalized therapeutic strategies. To this end, parameters such as stroke-work loss, valvular-arterial impedance, or ventriculo-arterial coupling have been investigated. Stroke-work index (SWI) loss is defined as the wasted LV energy due to valvular obstruction and increased afterload, calculated as the ratio of the mean Doppler-derived aortic gradient to the mean LV systolic pressure. HTN can lead to an underestimation of this index and, consequently, of the severity of the AS [24]. Valvulo-arterial impedance (Zva) estimates LV afterload as the sum of systolic BP and mean transvalvular gradient related to indexed stroke volume. Zva increases significantly with elevated BP and can favor an earlier onset of symptoms at a lower degree of stenosis than in normotensive patients. It has shown prognostic significance, even in patients after the transcatheter aortic valve replacement (TAVR) procedure [25,26,27,28]. Ventricular-arterial coupling (Ea/Ees) refers to the dynamic interaction between the LV and the arterial system, evaluating their efficiency in pumping blood and maintaining CV function. It is calculated as the ratio of arterial elastance and LV end-systolic elastance. For the same severity of aortic stenosis, this parameter is much more modified than in the presence of HTN [29]. However, these parameters have primarily been applied in research contexts and are therefore not explained in more detail in this review.

Cardiac magnetic resonance imaging (CMR) can provide additional information about LV remodeling and dysfunction caused by AS. Recent results have highlighted a distinct feature of LV remodeling due to AS, characterized by a shift in the LV axis (with the apex shifting to the right), compared with LV hypertrophy caused solely by high BP [20,30]. Moreover, increased extracellular volume fraction, as visualized by late gadolinium enhancement (LGE), in combination with serum biomarkers such as transforming growth factor beta 1 (TGF-β1), is a potent marker of fibrosis in patients with AS and another strong predictor of an adverse outcome [31,32].

High BP impairs the echocardiographic assessment of AS severity when flow-related criteria are used. High vascular resistance is correlated with lower transvalvular pressure gradients, lower flow rates, and lower stroke and cardiac indices [33]. Atrial fibrillation, significantly associated with HTN, can also influence the evaluation of AS severity, especially if it evolves at a high atrioventricular rate [7,17]. That is why current guidelines recommend rigorous control of BP and heart rate for a reliable estimate of AS severity, using both transvalvular gradients and a continuous equation to estimate aortic area [7,10,17].

Patients with AS and HTN have a significantly higher CV risk compared to normotensive patients with AS. After 4.3 years of follow-up in asymptomatic AS patients, high BP increased the incidence of LV hypertrophy by 51% and the risk of ischemic CV fatal events by 56%, and doubled the risk of mortality [1]. Similar results were found in a study with a 6.2-year follow-up period, in which CV mortality tripled, and all-cause mortality doubled in patients with AS and HTN compared with normotensive AS patients [23,34].

The treatment strategy for high BP in patients with AS involves, in the first phase, establishing optimal BP values. The SEAS study (The Simvastatin Ezetimibe in Aortic Stenosis study) has shown that in patients with asymptomatic AS, the optimal BP is 130–139 mmHg for systolic and 70–90 mmHg for diastolic BP [23]. Whether BP targets should be lowered further, as recommended in the 2024 ESC guideline for the treatment of high BP in patients at high CV risk, remains to be addressed in future studies [35].

Renin–angiotensin–aldosterone (RAAS) inhibitors are preferred over other antihypertensive medication classes in patients with AS and HTN. The most compelling data in favor of this choice come from studies of patients who underwent transcatheter aortic valve replacement (TAVR). Research has shown that angiotensin-converting enzyme inhibitors (ACEi) and angiotensin receptor blockers (ARBs) can reduce mortality for at least two years after a TAVR procedure, provided they were part of the treatment for high BP before the valve repair procedure [36,37]. The benefit of RAAS inhibitors is dose-dependent [38]. A debate that also awaits an answer from future research is the comparison of the benefits of ACE inhibitors versus ARBs, not only in lowering BP levels but also in slowing the progression of AS. Hypotheses support ARBs, suggesting that they may reduce inflammation, fibrosis, and calcification of the aortic valve by inhibiting aortic valve chymase and cathepsin G; however, they have not yet been clinically tested [39,40].

**Figure 1:**
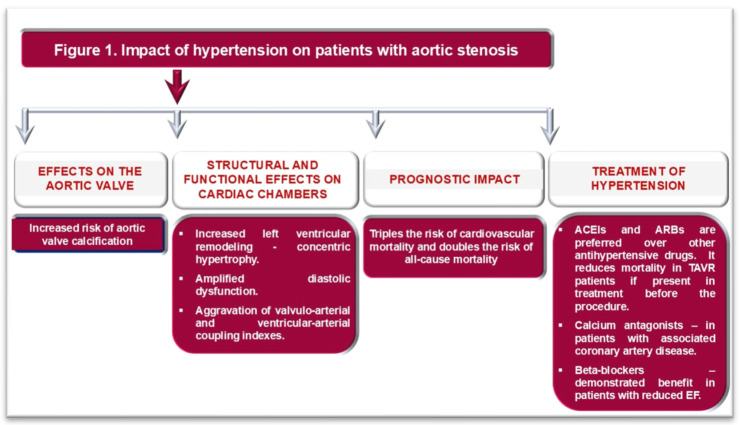
Impact of hypertension on patients with aortic stenosis. ACEIs: angiotensin-converting enzyme inhibitors; ARBs: angiotensin receptor blockers; EF: Ejection fraction of the left ventricle.

Beta-blockers demonstrated benefit after TAVR only in AS patients with reduced EF [41], whereas in those with preserved EF, their role remains controversial [23,33,35]. In patients with AS, HTN, and coronary artery disease, calcium antagonists may provide a better prognosis after TAVR [42]. The impact of HTN on the aortic valve, cardiac structure and function, outcomes, and the choice of appropriate hypotensive treatment in patients with AS are illustrated in Figure 1.

## 3. Aortic Regurgitation

Aortic valve regurgitation has a variable prevalence, ranging from 0.55 to 8.5% in women and 13% in men in the US or UK population, with a much lower prevalence of moderate and severe valve regurgitation (<2%) [43,44]. AR has the same determinants as AS, with the addition of male sex and autoimmune diseases (scleroderma, rheumatoid arthritis, systemic lupus erythematosus, Raynaud’s syndrome) [44]. The average age at diagnosis of AR is earlier than that of AS, around 57 years [4].

HTN contributes to the risk of both AR and AS. Each 20 mmHg increase in systolic BP is associated with a 1.38 times higher risk for the occurrence of AR [4]. This risk doubles when systolic BP exceeds 160 mmHg [4]. Although it has been hypothesized that aortic root dilatation may be the primary mechanism of AR in hypertensive patients, mounting data are beginning to point to the direct effect of BP on aortic valves [4,45]. There are different patterns of LV remodeling depending on the severity of AR and the presence of HTN. Eccentric hypertrophy characterizes the LV phenotype in HTN patients with moderate or severe AR, whereas concentric hypertrophy is present in HTN patients with mild AR [46]. Clinically significant AR is characterized by high volume preload and afterload, secondary eccentric LV hypertrophy, elevated PP, and isolated systolic HTN [46]. With the decrease in diastolic BP, myocardial hypoperfusion might occur [46]. This effect, together with the correlation between regurgitant volume and diastolic duration, explains why resting heart rate and diastolic BP are strong predictors of all-cause mortality in chronic moderate-to-severe AR, independent of medical and surgical treatment, and are associated with HTN [46,47]. While the relationship between mortality and resting heart rate is linear and proportional, with a cutoff at 60 bpm, diastolic BP shows an inverse, nonlinear relationship with mortality, resembling the tail of a J-shaped curve [47]. The risk becomes significant at diastolic BP values below 70 mmHg, peaking at 55 mmHg [47]. Furthermore, after surgical aortic valve replacement, only resting heart rate, but no diastolic BP, remains a significant predictor of CV mortality and correlates with reverse LV remodeling [47].

HTN amplifies the effect of AR on LA and LV remodeling and dysfunction. GLS is lower in patients with AR and HTN than in those with HTN alone [48]. With the progression of AR to the severe stage, a decrease in radial LV strain in the apical segment, an increase in LA volume, E/e’, and tricuspid regurgitation can be observed [49]. These findings are associated with reduced left atrial reservoir and conduit strain [48,49].

Due to its accuracy in assessing cardiac chambers, CMR has also shown that patients with AR and HTN have higher LV mass and end-diastolic volume than patients with HTN alone [46]. The presence of a bicuspid aortic valve leads to even greater diastolic and systolic LV volumes, reduced EF, and an increased proportion of LGE in CMR imaging [50]. Moreover, a bicuspid aortic valve is a significant risk factor for myocardial fibrosis, independent of HTN, LV mass, or volumes [50].

The prognostic impact of the combination of AR and HTN is best reflected in two remodeling patterns: the concentric phenotype, influenced chiefly by high BP, associated with an increased risk of stroke and coronary artery disease, whereas eccentric hypertrophy, mainly resulted from moderate or severe AR, correlates with the extension of myocardial fibrosis and with a high risk of ventricular arrhythmias [51,52].

**Figure 2:**
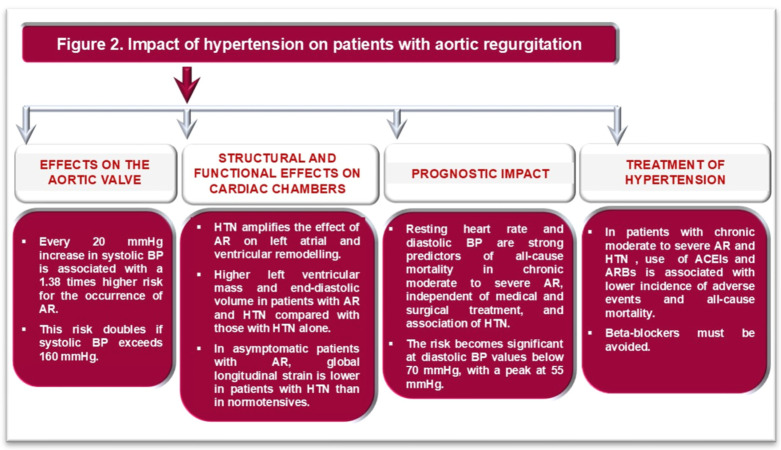
Impact of hypertension on patients with aortic regurgitation. ACEIs: angiotensin-converting enzyme inhibitors; ARBs: angiotensin receptor blockers; BP: blood pressure; HTN: arterial hypertension.

As in AS, the use of ACEi and ARBs in patients with chronic moderate to severe AR and HTN is associated with a lower incidence of adverse CV events and all-cause mortality [10,35]. In patents with AR and HTN, beta-blockers can be administered with caution [10]. On the one hand, they can amplify the wide PP in patients with AR, leading to higher isolated systolic BP values; on the other hand, they can affect diastolic duration, which might increase regurgitant volume [10]. The effects of HTN on the occurrence of AR and its severity, as well as on the adapted hypotensive treatment in these patients, are summarized in Figure 2.

## 4. Mitral Stenosis

The prevalence of mitral stenosis (MS) is about 12% among all patients with VHD [53]. Currently, rheumatic MS has a low incidence, while degenerative MS is often diagnosed in older people in developed countries [53]. MS is associated with various CV pathologies (74% hypertension, 58% coronary artery disease, 52% heart failure) and extracardiac disorders (anemia and chronic kidney disease) [54].

Even though MS does not directly influence LV function, associated HTN may exacerbate symptoms and aggravate MS by increasing the pressure gradient across the valve, particularly with high cardiac workload, and elevating pulmonary pressure [9,10,35]. Hypertensive patients with significant MS and preserved LVEF have decreased GLS and impaired LV myocardial work parameters compared to healthy subjects [55]. MS leads to autonomic nervous system dysfunction, characterized by hyperactive sympathetic tone, resulting in reduced cardiac output, increased atrial stretch, and altered BP circadian rhythm with reduced dipping [56]. Previous studies have demonstrated that normotensive patients with rheumatic MS had a higher prevalence of “non-dipping” BP pattern compared to healthy controls [56]. The clinical significance of this observation is important because a non-dipping profile is associated with an increased risk of CV disease, organ damage, and mortality [57,58].

Therapy for HTN in MS patients includes ACEi, ARBs, or dihydropyridines to achieve a systolic BP below 130 mmHg [35]. Acute heart failure in MS patients requires diuretics carefully titrated to avoid worsening of renal function, hypotension, and dehydration [35].

## 5. Mitral Regurgitation

Mitral regurgitation (MR) has a high prevalence in the general population, up to 10%, if mild or even trivial forms are included [59,60]. Rates of “moderate” or “severe” MR are much lower, at up to 2% [60]. It is important to note that HTN might amplify the risk of MR, and this risk increases significantly with age [2,60]. Each 20 mmHg increase in systolic BP and each 10 mmHg increase in diastolic BP amplify the risk of developing MR by 26% and 24% respectively [2]. Furthermore, women show a stronger correlation between HTN and MR than men, at the expense of other CV risk factors such as dyslipidemia, diabetes, and renal dysfunction [61,62]. In addition, hypertriglyceridemia in women increases the risk of developing MR, likely due to the high risk of mitral annular calcification [62]. Primary MR develops more frequently in women, while men have an increased risk of secondary MR attributable to higher alcohol and tobacco consumption [59]. In addition, 87% of the effect of HTN on MR is independent of the LV’s background pathology, suggesting a direct effect of BP on the mitral valve apparatus [2].

Chronic MR, regardless of etiology (primary or secondary), results in volume overload, increased myocardial stress, and LV dilation [60]. In addition, HTN amplifies afterload and promotes LV dysfunction and remodeling through this mechanism [60]. Geometric changes in the LV cavity lead to tethering of the mitral valve annulus and leaflets, as well as displacement of the papillary muscles, thereby worsening MR [60].

The pathogenic relationship between primary MR and HTN is complex and inconclusive [60]. In systole, HTN increases LV pressure and mechanical stress on the mitral valve apparatus [61,62]. In diastole, increased LA pressure exerts a greater effect on both the atrial and ventricular valvular surfaces [62,63]. Thus, HTN can trigger or worsen intrinsic valvular degeneration [60]. Furthermore, persistently elevated BP exerts a damaging hemodynamic effect on an insufficient MV, increasing the regurgitant orifice area and regurgitant flow [60,64]. Moreover, calcification of the mitral annulus or leaflets shares a common background with atherosclerosis, for which HTN is a significant risk factor [60,65]. HTN is strongly associated with idiopathic rupture of the mitral chordae tendineae (CTR) [66]. Long-term HTN induces high mechanical shear stress on the mitral valve apparatus, including chordae tendineae [66,67]. Hypertensive patients with CTR exhibit high expression of tissue inhibitor of metalloproteinase-2, a trigger of cardiac fibrosis, leading to a 10-fold increase in CTR compared with only a 3-fold increase in CTR from other etiologies [68].

Secondary MR, a heterogeneous condition with atrial and ventricular phenotypes, is not a valve disease per se, but a consequence of an imbalance between closing and tethering forces on the mitral valve apparatus [69,70]. High BP increases the risk of coronary artery disease and heart failure, diseases that can lead to the development of secondary MR due to LV remodeling [59,60]. The pathogenic mechanisms underlying secondary MR include papillary muscle dysfunction and mitral annulus dilatation, with loss of the mitral valve’s saddle shape [60,71]. Ischemic MR that occurs following a myocardial infarction, usually in the inferoposterior territory, is characterized by the posterior and lateral displacement of the papillary muscles, resulting in an incomplete MV closure and altered coaptation [64]. Nonischemic MR, secondary to LV global dysfunction and remodeling, is characterized by apical displacement of papillary muscles and leaflet tethering [64]. As in the case of primary MR, HTN can constitute an additional factor in ventricular and atrial remodeling, with impaired leaflet coaptation and mitral valve tenting [72]. Atrial fibrillation can worsen this vicious cycle between MR and structural or functional alterations of the left atrium, including the so-called “stiff LA syndrome” [60,72,73,74,75]. As proof, restoration of sinus rhythm may improve functional MV by LA and reverse remodeling of the mitral valve apparatus [60,74,75].

In patients with HTN and MR, EF is maintained for a long time, but myocardial deformation begins to decrease even in patients with mild MR [75]. It progressively decreases with increasing MR severity [75]. Thus, GLS, not EF, is an independent predictor of pulmonary hypertension and survival in patients with MR [75,76]. MR leads to an increase in LV mass and volume, as well as a decrease in GLS compared to healthy individuals or patients with HTN alone [76,77]. The MR degree and LV deformation are powerful independent predictors of LA function. Ventricular dysfunction, in this context, becomes the third link in the amplification of MR and LA dysfunction [77]. Parameters of systolic LV function are strongly associated with high preload, assessed by LA volume and strain [75]. Thus, HTN with mild MR leads to an increase in LA volume and a decrease in reservoir function; moderate MR develops an additional reduction in LA pumping and conduction function; while severe MR is characterized by a significant increase in LA volume and significant impairment of its function [77].

Medical treatment of HTN in MR patients includes RAAS inhibitors, beta-blockers, and vasodilators to reduce BP below 130/80 mmHg [10,35,60]. Antihypertensive drugs have additional beneficial effects on the mitral valve apparatus by reducing mitral valve calcification and shear forces, on the left heart function by lowering LV wall stress, LA preload, and pressure, and on the severity of MR by reducing regurgitant volume and orifice area [9,60].

In patients with primary MR and HTN, RAAS inhibitors may decrease LA and LV volumes and mass and improve MR severity [60,78]. Similar effects are observed for beta-blockers [79]. In addition to favorable clinical effects, including reductions in NYHA class and brain natriuretic peptide level, beta-blockers reduce MR severity and favor LV reverse remodeling [80]. However, data are extracted from small, non-randomized, and heterogeneous studies with no consistent report of clinical outcomes [60]. Therefore, no firm recommendation can be made about the benefit of antihypertensive medication on the severity of the valvular lesion, LA and LV remodeling, pulmonary hypertension, and outcome [10,35,60]. There is a substantial gap in the evidence on the effects of antihypertensive treatment in patients with MR, and studies evaluating optimal BP targets under treatment and the impact of different drug classes remain an open field of research.

**Figure 3:**
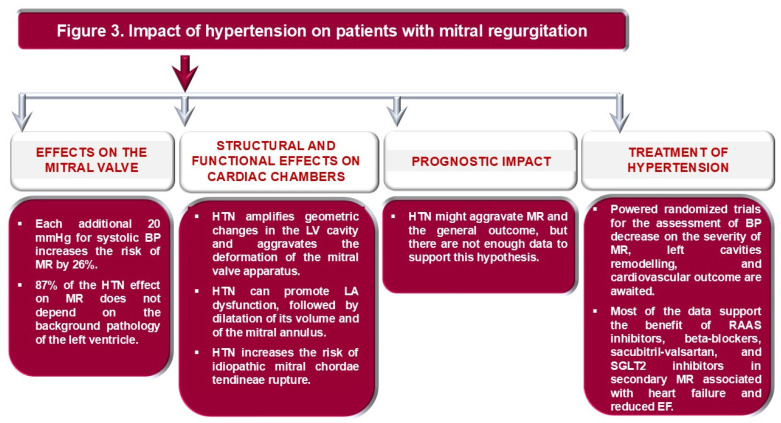
Impact of hypertension on patients with mitral regurgitation. BP: blood pressure; EF: ejection fraction of the left ventricle; HTN: arterial hypertension; LA: left atrial; LV: left ventricle; MR: mitral regurgitation; RAAS: renin–angiotensin–aldosterone system; SGLT2 inhibitors: inhibitors of sodium-glucose cotransporter 2 in the kidneys.

On the contrary, patients with secondary MR and HTN with heart failure or coronary heart disease benefit from antihypertensive therapy, which also has a positive effect on mitral valve function and prognosis [60]. Beta-blockers, especially when LVEF is below 40%, not only reduce the severity of MR but also improve LV remodeling and increase EF [60,80]. RAAS inhibitors have a similar effect on the heart [81]. Beyond that, RAAS inhibitors and beta-blockers reduce mortality and correlate with improved event-free survival [81]. However, in hypertensive patients with secondary MR due to ischemic heart disease, early administration of sacubitril-valsartan after coronary artery revascularization reduces MR severity, improves LV systolic function, and survival [82]. Likewise, SGLT2 inhibitors ameliorate MR severity and improve cardiac function, with a significant decrease in LV and LA deformation [10,83]. Diuretics and vasodilators attenuate MR and ameliorate impaired preload and afterload, with a symptomatic role in congestive heart failure [59]. The effects of HTN on the mitral valve, the structure and function of cardiac chambers, and medical treatment, with possible impact on prognosis in MR, are summarized in Figure 3.

## 6. Conclusions

HDV and HTN frequently coexist, especially in the elderly and in patients with aortic stenosis. Still, this association does not result only from their concomitant manifestation, but also from the direct impact of elevated BP on the structure and function of the left heart valves. Extensive epidemiological studies have proven that high BP is the leading modifiable CV risk factor in VHD patients.

There is a strong interplay between VHD and HTN. HTN remodels the left cardiac chambers and increases their filling pressures. These effects on the architecture of the valvular apparatus or transvalvular pressure gradients, specific to one type of valvular lesion, aggravate VHD and associated heart failure, leading to worsening the prognosis. In advanced stages, BP values are, in turn, influenced by valvulopathies. In AS, high BP aggravates LV hypertrophy and indices of valvulo-arterial and ventricular–arterial coupling, thereby increasing the LV workload. In AR, higher LV mass and end-diastolic volumes have been found in the presence of HTN than in those with normal BP. In AR, beta-blockers should be avoided. MR is a very heterogeneous valvular lesion. In secondary MR, HTN is also found among the conditions favoring coronary artery disease or heart failure, increasing the likelihood of its occurrence. Still, it is recognized as an aggravating factor, a change in LV geometry leading to tethering of the mitral valve annulus and leaflets, as well as displacement of the papillary muscles and the dilatation of the LA, which further increases the mitral annulus.

In evaluating patients with VHD, we must take into account BP values that can favor the misclassification of their severity. In addition, new echocardiographic and cardiac MRI techniques aim to distinguish the contribution of HTN from that of VHD to cardiac damage, enabling more individualized therapy and better determining the timing of valvular correction.

There are no large randomized controlled trials examining the role of antihypertensive medications in the progression of various VHDs. The target BP under antihypertensive treatment is not yet established: for AS, the values might be, with high probability, 130–139 mmHg for systolic and 70–90 mmHg for diastolic BP; for AR, it is only known that the risk becomes significant at diastolic BP values below 70 mmHg, peaking at 55 mmHg, but still it is not established an optimal interval of values; for MR, the BP target has been identified to be lower than 130/80 mmHg. Regarding the classes of drugs indicated, there is a clear benefit of ACEs and ARBs proven for aortic valve pathology. In secondary MR, there is evidence of improvement in severity with treatment for heart failure and reduction in left chambers remodeling with ACE inhibitors/ARBs, sacubitril/valsartan, and SGLT2 inhibitors. However, there is a substantial gap in the evidence on the effects of antihypertensive treatment in patients with primary MR, and studies evaluating optimal BP targets under treatment and the impact of different drug classes remain underexplored.

## Data Availability

The original contributions presented in the study are included in the article, further inquiries can be directed to the corresponding author.

## Abbreviations

ACEi	angiotensin-converting enzyme inhibitors
AR	aortic regurgitation
ARB	angiotensin-receptor blockers
AS	aortic stenosis
BP	blood pressure
CMR	cardiac magnetic resonance
CTR	mitral chordae tendineae rupture
CV	cardiovascular
EF	ejection fraction
GLS	global longitudinal strain
HTN	hypertension
LA	left atrium
LGE	late gadolinium enhancement
LV	left ventricle
MR	mitral regurgitation
MS	mitral stenosis
PP	pulse pressure
RAAS	renin–angiotensin–aldosterone system
SWI	stroke-work index
TAVR	transcatheter aortic valve replacement
VHD	valvular heart disease
Zva	valvulo-arterial impedance
